# Targeting Notch1 and proteasome as an effective strategy to suppress T-cell lymphoproliferative neoplasms

**DOI:** 10.18632/oncotarget.3621

**Published:** 2015-04-02

**Authors:** Lujun Yang, Shuangfeng Zhang, Suraj Konnath George, Rong Teng, Xuefen You, Mengqi Xu, Hong Liu, Xiaoping Sun, Hesham M. Amin, Wenyu Shi

**Affiliations:** ^1^ Department of Hematology, Affiliated Hospital of the University of Nantong, Jiangsu 226001, China; ^2^ Department of Hematopathology, The University of Texas MD Anderson Cancer Center, Houston, Texas 77030, USA; ^3^ Department of Laboratory Medicine, The University of Texas MD Anderson Cancer Center, Houston, Texas 77030, USA; ^4^ The University of Texas Graduate School of Biomedical Sciences, Houston, Texas, 77030, USA

**Keywords:** T-cell lymphoproliferative neoplasms, Notch1, proteasome, γ-secretase inhibitors, bortezomib

## Abstract

The T-cell lymphoproliferative neoplasms (T-LPN) are characterized by a poor clinical outcome. Current therapeutics are mostly non-selective and may induce harmful side effects. It has been reported that *NOTCH1* activation mutations frequently associate T-LPN. Because anti-Notch1 based therapies such as γ-secretase inhibitors (GSI) are less efficient and induce considerable side effects, we hypothesized that combining low concentrations of GSI and the proteasome inhibitor bortezomib (BTZ) may provide an effective and tolerable approach to treat T-LPN. Hence, we analyzed the *in vitro* and *in vivo* effects of GSI-I and BTZ, alone or in combination, against T-LPN. GSI-I and BTZ synergistically decreased cell viability, proliferation, and colony formation, and induced apoptosis in T-LPN cell lines. Furthermore, combining GSI-I and BTZ decreased the viability of primary T-LPN cells from patients. These effects were accompanied by deregulation of Notch1, AKT, ERK, JNK, p38 MAPK, and NF-κB survival pathways. Moreover, combination treatment inhibited T-LPN tumor growth in nude mice. In all experiments, combining low concentrations of GSI-I and BTZ was superior to using a single agent. Our data support that a synergistic antitumor activity exists between GSI-I and BTZ, and provide a rationale for successful utilization of dual Notch1 and proteasome inhibition to treat T-LPN.

## INTRODUCTION

The T-cell lymphoproliferative neoplasms (T-LPN) constitute a heterogeneous group of aggressive hematopoietic malignancies with limited chemotherapeutic options that are frequently incapacitated by side effects, chemoresistance, relapse, and poor clinical outcome [1, 2]. Despite that intense chemotherapy has marginally improved treatment efficacy, only transient responses accompanied with deleterious and life-threatening side effects have been achieved.

Notch signaling plays crucial roles in normal cellular homeostasis; nonetheless, a growing body of evidence suggests that it also influences intricate phases of tumorigenesis such as cell proliferation, metabolism, growth, and survival [3–7]. Notch receptors are essentially synthesized as pre-Notch in the endoplasmic reticulum, which is then cleaved by Furin-like convertase resulting in a heterodimeric receptor with non-covalently associated domains that is transported from the Golgi network to the plasma membrane [8]. The Notch signaling network primarily involves 5 cell membrane-based ligands – Jagged 1 (JAG1), JAG2, Delta-like ligand 1 (DLL1), DLL3, and DLL4 – each of which binds and activates the Notch receptors (Notch1–4) of neighboring cells. Ligand binding interactions induce enzymatic cleavage of the Notch1 receptor by metalloproteinase and γ-secretase resulting in the release of an intracellular fragment of Notch1 known as Notch1 intracellular domain (NICD), which subsequently translocates to the nucleus to activate the transcription of downstream target genes [9, 10]. For the transcription regulation across different species, the activation of the Notch pathway can also be accomplished in a non-canonical manner irrespective of ligand-induced cleavage [11–14].

Notch1 has distinct physiologic roles in lineage commitment, differentiation, and function of normal T lymphocytes, yet it has also been associated with survival promoting effects in T-LPN. It has been reported that the reciprocal chromosomal translocation t(7;9)(q34;q34.3), which involves the *NOTCH1* and the T-cell receptor-β (*TCRB*) genes, occurs in T lymphoblastic leukemia/lymphoma (TLL) [15]. Moreover, approximately 50% of TLL patients harbor mutations that cause *NOTCH1* constitutive activation [16]. These observations suggest the involvement of Notch1 in T-cell oncogenesis. Therefore, blockade of Notch1 signaling by the γ-secretase inhibitors (GSI) has emerged as a promising therapeutic strategy to suppress T-LPN. GSI not only have cytostatic effects but also induce apoptosis in T-LPN [16–19]. Alas, phase I clinical trials using GSI have reported gastrointestinal toxicity in the form of intractable diarrhea and increased goblet cell differentiation associated with intestinal secretory metaplasia, which threatens the feasibility of this approach to treat cancer patients [20, 21].

Recently, proteasome inhibition has been evolving as a potential therapeutic approach for a variety of cancers including hematological malignancies [22–26]. The ubiquitin-proteasome pathway is actively involved in intracellular protein turnover, which controls cellular homeostasis. Because the majority of cancer cells exhibit higher levels of proteasome activity, they are more prone to the negative effects of proteasome inhibitors such as bortezomib (BTZ, Velcade), a reversible proteasome inhibitor that has been approved by the FDA to treat subtypes of hematological malignancies including plasma cell myeloma and mantle cell lymphoma [24, 27]. Nonetheless, dose-limiting toxicity including peripheral neuropathy represents a major drawback for the utilization of proteasome inhibitors in clinical settings [28].

Because of the limitations that hinder using Notch1 and proteasome inhibitors as single agents to treat T-LPN, we hypothesized that combining low concentrations of Notch1 and proteasome inhibitors may prove to be a safer and perhaps more superior strategy to suppress T-LPN than using higher concentrations of each of these inhibitors alone. To achieve our goals, we performed comprehensive *in vitro* and *in vivo* characterizations of the single and combined antitumor effects of the γ-secretase inhibitor GSI-I and the proteasome inhibitor BTZ in T-LPN. Our data support that these two drugs interact in a synergistic fashion to induce cell death and inhibit the proliferation of T-LPN, which are associated with remarkable perturbations in cell survival regulatory proteins. Importantly, the GSI-I and BTZ combined regimen successfully reduces T-LPN tumor size in a murine xenograft model. Our results suggest that this novel strategy could be successfully utilized to treat T-LPN patients in the future.

## RESULTS

### Combined treatment with GSI-I and BTZ induces apoptosis and decreases the proliferation and anchorage-independent colony formation of T-LPN

**Figure 1:**
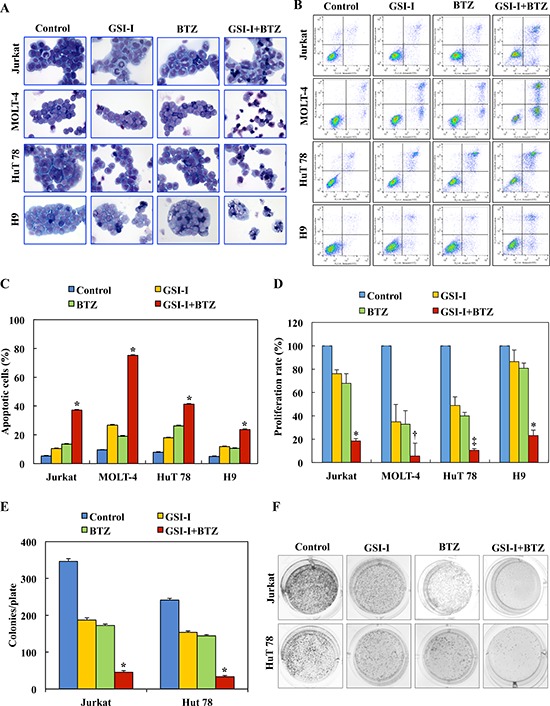
Combined treatment with GSI-I and BTZ induces apoptosis and decreases the proliferation and anchorage independent colony formation of T-LPN cells **A.** Giemsa staining shows that treating T-LPN cells with GSI-I or BTZ alone induced mild increase in apoptotic cells. Combined treatment by GSI-I and BTZ was much more effective in inducing apoptosis in T-LPN cells. Jurkat and H9 cells were the least and most sensitive to the effects of the combined treatment. Morphological features consistent with apoptosis included cellular shrinkage, cytoplasm vacuolization, and nuclear condensation and fragmentation (original magnification: ×400). **B.** Examples of flow cytometry dot plots showing that, compared with control untreated T-LPN cells, the Annexin V-positive cells (right upper and lower quadrants) are remarkably increased after combined treatment with GSI-I and BTZ than after treatment with a single agent. **C.** Although GSI-I or BTZ caused cell apoptosis when used as single agents, the effects of combined treatment was more robust (*: *P* < 0.0001 compared with control, GSI-I, and BTZ). **D.** GSI-I or BTZ as a single agent decreased the proliferation of T-LPN cells, with MOLT-4 and HuT 78 demonstrating more pronounced effects than Jurkat and H9 cells. However, combined treatment by GSI-I and BTZ was much more effective in reducing cellular proliferation than any of the two inhibitors alone (*: *P* < 0.001 vs. control and *P* < 0.05 vs. GSI-I and BTZ; †: *P* < 0.0001 vs. control, GSI-I, and BTZ; ‡: *P* < 0.0001 vs. control and GSI-I and *P* < 0.001 vs. BTZ). **E.** Whereas GSI-I or BTZ decreased the anchorage-independent colony formation of Jurkat and HuT 78 cells in methylcellulose, their combined effects were much more dramatic (*: *P* < 0.0001 compared with control, GSI-I and BTZ). **F.** Representative examples of the colonies after treatment with different regimens. Results shown in C, D, and E represent the means ± SE of three independent experiments.

Compared with a single agent, treatment of T-LPN cell lines with a combination of GSI-I and BTZ for 24 h caused more pronounced apoptosis as illustrated by characteristic morphological features including cell shrinkage, cytoplasmic vacuolization, and nuclear condensation and fragmentation (Fig. 1A). The number of apoptotic cells as defined by the morphological criteria varied among the different cell lines, with H9 and Jurkat cells demonstrating the highest and lowest numbers of apoptotic cells, respectively. Moreover, flow cytometric analysis using Annexin V-FITC/PI dual staining showed that higher percentage of T-LPN cells underwent apoptosis in response to the combination treatment than the individual drugs (Fig. 1B and 1C). In addition, at 24 h, cell proliferation measured by BrdU assay, was significantly decreased in response to the combination treatment compared to the single agent (Fig. 1D). A clonogenic assay was also performed to assess individual and combined effects of GSI-I and BTZ on T-LPN anchorage-independent colony formation. Whereas GSI-I or BTZ alone decreased colony numbers, the combined treatment caused more reduction in the number of HuT 78 and Jurkat cells colonies (Fig. 1E). Images of representative colonies from different treatment groups are shown (Fig. 1F).

### Combined treatment with GSI-I and BTZ induces significant apoptosis and decreases the viability of primary T-LPN cells, but not normal human T lymphocytes

**Figure 2:**
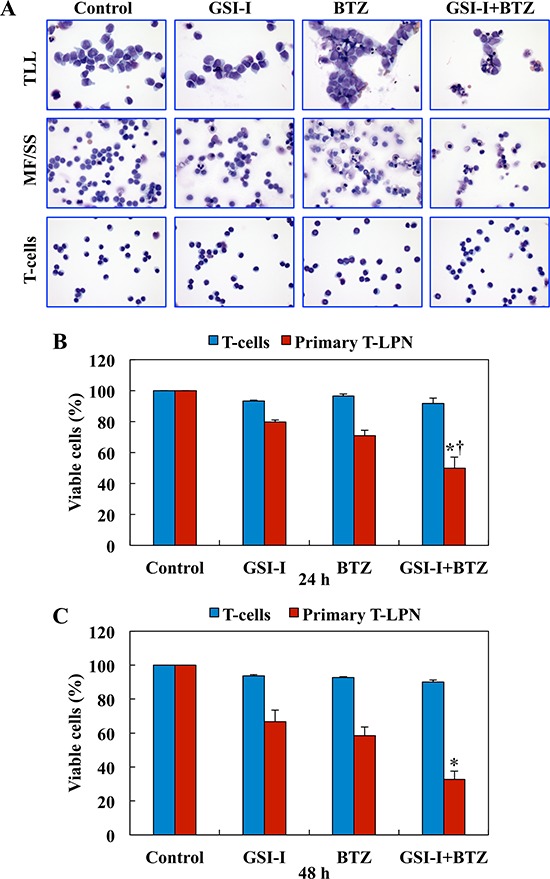
Combined treatment with GSI-I and BTZ induces apoptosis and decreases the viability of primary T-LPN, but not of normal human T lymphocytes **A.** Microscopic analysis of primary T-LPN cells from patients treated *ex vivo* with GSI-I (2.5 μM) alone or combined with BTZ (10 nM) for 24 h and stained with Giemsa showed morphological features of apoptosis to be more pronounced after the combination treatment than the single drug treatment. Examples from TLL and MF/SS cases are shown (original magnification: ×400). Combination treatment with GSI-I and BTZ also decreased the viability of primary T-LPN cells collected from patient samples (*N* = 8) at **B.** 24 h (*: *P* < 0.0001 vs. control and GSI-I and †: *P* < 0.01 vs. BTZ) and **C.** 48 h (*: *P* < 0.0001 vs. control, GSI-I, and BTZ). Importantly, the viability of human T lymphocytes was not decreased after the treatment. Data shown represent the means ± SE of 8 different samples.

We studied the effects of treatment with GSI-I and BTZ on primary T-LPN cells obtained from 8 patients (4 females and 4 males, with a median age of 43.5 years). The patients' specimens included 1 bone marrow aspirate and 7 peripheral blood samples. Five patients had TLL (median age: 28 years), and 3 had mycosis fungoides/Sèzary syndrome (MF/SS; median age: 68 years). *NOTCH1* mutations were detected in 3 of 4 TLL patients. Mutation analysis was not performed in the remaining patients. Representative photomicrographs are shown after treatment with GSI-I or BTZ, alone or in combination (Fig. 2A). The apoptotic effects induced by combined treatment were more pronounced than the effects caused by a single agent. Importantly, apoptosis was not observed in normal human T lymphocytes treated with GSI-I and BTZ, and used as a control. Moreover, compared with single agent treatments, combined treatment with GSI-I and BTZ was associated with a more pronounced decrease in the viability of primary human T-LPN cells at 24 h, and the treatment did not induce notable decrease in the viability of normal human T lymphocytes (Fig. 2B). Despite that the effects of the combined treatment with GSI-I and BTZ on T-LPN cells became more pronounced at 48 h, minimal effects were observed in the T lymphocytes (Fig. 2C).

### Combination of GSI-I and BTZ induces synergistic inhibitory effects in T-LPN

**Figure 3:**
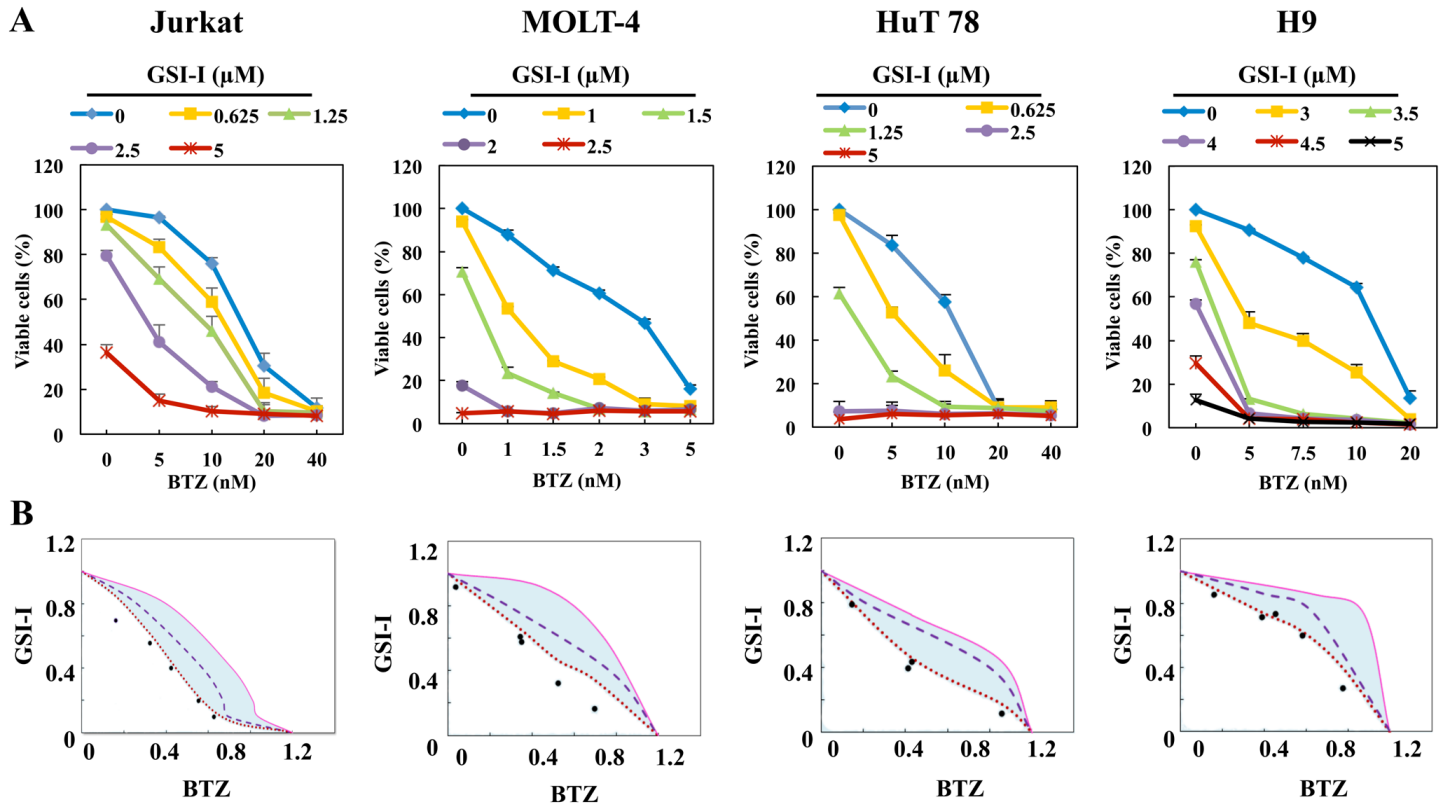
Combining GSI-I and BTZ induces synergistic inhibitory effects in T-LPN **A.** Compared with a single drug, combined treatment with GSI-I with BTZ, induced significant decrease in the viability of Jurkat, MOLT-4, HuT 78, and H9 cells at 48 h. Data represent means ± SE from three independent experiments. **B.** Isobolographic curves illustrate that majority of the data points are lying to the left of the envelope of additivity (light blue area), indicating that combining GSI-I and BTZ induces synergistic effects in T-LPN cells.

Because the inhibitory effects were more pronounced when GSI-I and BTZ were simultaneously used to treat T-LPN than when a single agent was used alone, we set to examine whether the potentiation of the effects of combined treatment resulted from synergistic or additive interactions between GSI-I and BTZ. The TLL cell lines, Jurkat and MOLT-4, and the MF/SS cell lines, HuT 78 and H9, were treated with different concentrations of GSI-I and/or BTZ for 48 h. Treatment using GSI-I or BTZ alone resulted in a concentration-dependent decrease in cell viability of T-LPN cells. Nevertheless, the combination treatment was superior in decreasing T-LPN cell viability compared with single agents. For example, 1.25 μM GSI-I or 10 nM BTZ induced approximately 40% reduction in HuT 78 cell viability, but in combination the same concentrations induced more than 80% decrease in the viability of HuT 78 cells (Fig. 3A). To evaluate whether the combined effects of GSI-I and BTZ are synergistic or additive, isobologram analysis was performed. The area surrounded by the isoeffect curves is referred to as the envelope of additivity (Fig. 3B). When the data points of the drug combination fall within the envelope, the combined effects are considered additive. When the data points fall to the left of the envelope, the combined effects are caused by low concentrations of the two agents than is predicted, and the combination effect is regarded as synergistic. Isobologram analysis showed that the majority of the data points are located to the left of the envelope, indicating synergistic interactions between GSI-I and BTZ.

### Combined treatment with GSI-I and BTZ induces pronounced alterations in survival pathways in T-LPN

**Figure 4:**
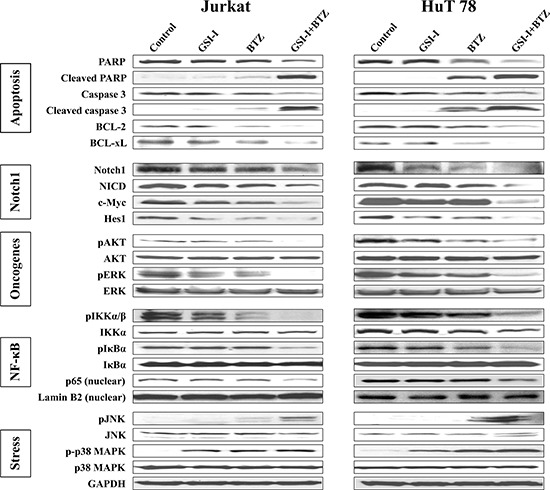
Combined inhibition with GSI-I and BTZ induces biochemical changes consistent with apoptosis and cell death in T-LPN Western blot analysis showed that treatment of Jurkat and HuT 78 cells simultaneously with GSI-I and BTZ induces cleavage of caspase-3 and PARP, and decreases BCL-2 and BCL-xL, which is consistent with the occurrence of apoptosis. The effects of the combined treatment were more pronounced than the effects of a single agent. Furthermore, Notch1 and NICD along with the Notch1 target proteins c-Myc and Hes1 were markedly decreased after the combination treatment in comparison with the single agent treatment. Compared with GSI-I or BTZ alone, the expression of pAKT and pERK were significantly downregulated when GSI-I+BTZ were used to treat the cells indicating more negative impact of the combination treatment on T-LPN cell survival. Combination strategy also reduced the levels of pIKKα/β, pIκBα, and nuclear p65 more than single agents. The levels of pJNK were upregulated after the combined treatment. However, only a slight increase in the levels of p-p38 MAPK was noted after treatment with GSI-I and BTZ.

To this end, we sought to explore the effects of GSI-I and BTZ on molecular pathways that facilitate T-LPN survival (Fig. 4). In Jurkat and HuT 78 cells, treatment with BTZ alone, but not GSI-I, slightly increased cleaved caspase-3 and cleaved PARP levels without noticeable effects on their total form levels. In addition, BCL-2 and BCL-xL were slightly decreased after treatment with BTZ alone. Nonetheless, the combination treatment with GSI-I and BTZ enhanced in a robust manner the decrease in total caspase 3 and total PARP, and the increase in their cleaved forms as well as the decrease in BCL-2 and BCL-xL. These biochemical changes are in agreement with the occurrence of apoptosis.

To evaluate Notch1 activity, we examined the expression of full-length Notch1, NICD, and the Notch1 targets c-Myc and Hes1. Individually, GSI-I or BTZ was able to slightly or moderately decrease Notch1 expression. In addition, the expression of NICD and Notch1 target proteins including c-Myc and Hes1 was also mildly downregulated after treatment with GSI-I or BTZ. The decrease in these proteins was markedly enhanced after using combined treatment with GSI-I and BTZ.

Next, we studied the effects of GSI-I or BTZ alone or in combination on AKT and ERK survival kinases (Fig. 4). In Jurkat cells, no notable changes were observed in pAKT after treatment with GSI-I or BTZ alone, whereas in HuT 78 cells each of the two agents minimally to moderately decreased the expression of pAKT. A marginal decrease in pERK was also detected in the two cell lines after treatment with GSI-I or BTZ alone. In contrast, the combination treatments abrogated the expression of pAKT and pERK in Jurkat cells, and substantially decreased their levels in Hut 78 cells. No changes were noticed in basal AKT and ERK.

Furthermore, NF-κB signaling proteins were analyzed after treatment with GSI-I and BTZ (Fig. 4). In whole cell lysates, GSI-I or BTZ mildly to moderately decreased the expression of pIKKα/β, with no obvious change in total IKKα levels. After treatment with the combined regimen, the levels of pIKKα/β decreased significantly. Whereas nuclear p65 expression remained largely unaffected after single agent treatments, combined GSI-I and BTZ downregulated p65 nuclear expression. Although changes were not noticed in pIκBα in Jurkat cells after a single agent treatment, a slight decrease in pIκBα expression was found in HuT 78 cells after treatment with BTZ. Notably, combined treatment with GSI-I and BTZ decreased significantly pIκBα levels without affecting the basal levels of IκBα. Treating the cells with BTZ was expected to inhibit the degradation of IκBα protein, which would lead to an increase in IκBα basal levels. Alternatively, in HuT 78 cells, BTZ decreased pIκBα, which is the inactivated form of IκBα. This decrease resulted in an indirect increase in the basal levels of IκBα through the increase in the IκBα-to-pIκBα ratio (33% increase based on densitometry studies of the IκBα and pIκBα WB bands; densitometry data are not shown). Of important note, combining BTZ and GSI-I remarkably increased basal IκBα protein (107% increase) relative to the substantial decrease in pIκBα levels. Similar effects on IκBα basal levels were noted in Jurkat cells when BTZ alone or combined with GSI-I was used (the increase in IκBα-to-pIκBα ratio was 21% and 74%, respectively).

The effects of GSI-I and BTZ on stress-related proteins were also studied (Fig. 4). Individually, each of the two drugs did not exert notable effects on pJNK levels, but the combined treatment remarkably enhanced JNK phosphorylation. In addition, the levels of p-p38 MAPK were moderately increased after treatment with GSI-I or BTZ alone, but the combined treatment slightly enhanced this increase.

### Individual contributions of AKT and p38 MAPK signaling to the synergistic effects of GSI-I and BTZ in T-LPN

**Figure 5:**
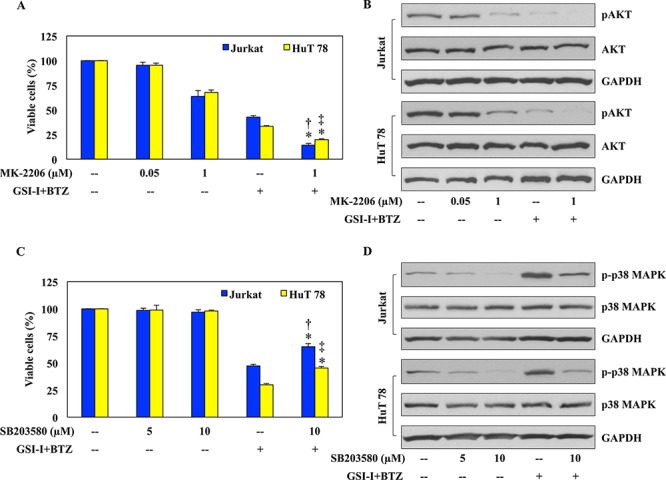
AKT and p38 MAPK contribute to the synergistic effects of GSI-I and BTZ in T-LPN **A.** Jurkat and HuT 78 cells treated with MK-2206 at 48 h showed a slight concentration-dependent decrease in their viability. The decrease in cell viability was more prominent after combined treatment with GSI-I and BTZ. However, in the presence of MK-2206, GSI and BTZ were able to further enhance their inhibitory effects on T-LPN cell viability. **B.** Western blot analysis showed a concentration-dependent decrease of pAKT after treating Jurkat and HuT 78 cells with MK-2206, and this decrease was more pronounced when a combination of GSI-I and BTZ were used. However, in the presence of MK-2206, GSI-I and BTZ completely abrogated the expression of pAKT. There were no changes in the basal levels of AKT proteins. GAPDH confirms equal protein loading. **C.** Jurkat and HuT 78 cells treated with SB203580 for 48 h demonstrated no significant changes in their viability. Although, combination treatment of GSI-I and BTZ drastically reduced cellular viability, this decrease was partially rescued in the presence of SB203580, GSI-I, and BTZ combined treatment. **D.** The expression of p-p38 MAPK demonstrated a concentration-dependent decrease after treatment with SB203580 alone. In contrast, the expression of p-p38 MAPK was remarkably increased after using GSI-I and BTZ to simultaneously treat the T-LPN cells. In the presence of SB203580, GSI-I, and BTZ collectively managed to partially rescue the expression of p-p38 MAPK. Changes were not observed in the basal levels of p38 MAPK protein. GAPDH was used as the loading controls. Data shown in (A) and (C) represent the means ± SE from three independent experiments (*: *P* < 0.001 vs. GSI-I + BTZ alone in both cells; †: *P* < 0.001 vs. MK-2206 [1 μM] or SB203580 [10 μM] in Jurkat; ‡: *P* < 0.0001 vs. MK-2206 [1 μM] or SB203580 [10 μM] in HuT 78).

To further decipher the individual contributions of AKT and p38 MAPK to the synergistic effects of GSI-I and BTZ, selective inhibitors were utilized followed by analysis of cell viability and protein expression. Treatment with the selective AKT inhibitor MK-2206 at 0.05 μM for 48 h did not decrease the viability of Jurkat and HuT 78 cells (Fig. 5A). The decrease in cell viability became slightly more pronounced when a concentration of 1.0 μM was used. Nonetheless, combining GSI-I and BTZ caused marked decrease in the viability of the T-LPN cells. Moreover, combining MK-2206, GSI-I, and BTZ further decreased T-LPN cellular viability in the two cell lines. At the biochemical level, the expression of pAKT was moderately reduced in Jurkat and HuT 78 cells treated with MK-2206 at a concentration of 1.0 μM for 24 h (Fig. 5B). The decrease in pAKT became more pronounced when GSI-I and BTZ were simultaneously used to treat the cells. Importantly, combining MK-2206 with GSI-I and BTZ completely abrogated the expression of pAKT. Total AKT levels remained unaffected throughout the experiments.

To characterize the role of p38 MAPK in the synergistic effects of GSI-I and BTZ, SB203580, a selective inhibitor of p38 MAPK, was used in a similar fashion (Fig. 5C). While SB203580 had negligible effects on cell viability at 5 and 10 μM concentrations, combined treatment with GSI-I and BTZ for 48 h remarkably decreased the viability of Jurkat and HuT 78 cells. These effects were partially rescued when SB203580 was simultaneously used with GSI-I and BTZ. Western blotting demonstrated that SB203580 induced a concentration-dependent decrease in p-p38 MAPK levels, and this decrease was reversed when the combination treatment of GSI-I with BTZ was used (Fig. 5D). Importantly, when SB203580 was additionally used to treat the cells with GSI and BTZ, the expression of p-p38 MAPK was decreased. Total p38 MAPK levels remained unchanged in response to the different treatments.

### Combined treatment with GSI-I and BTZ suppresses T-LPN xenograft tumor growth in nude mice

**Figure 6:**
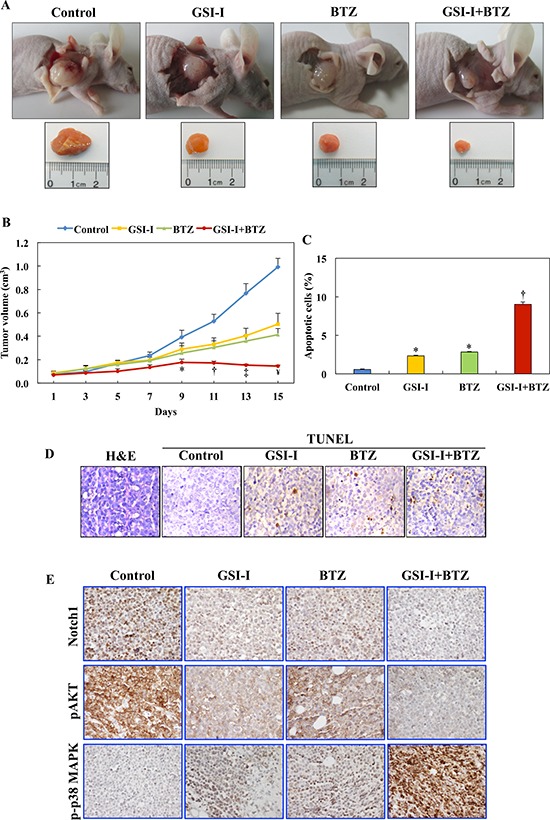
GSI-I and BTZ effectively suppressed T-LPN xenograft tumor growth **A.** In nude mice (examples shown) challenged with subcutaneous injection of Jurkat cells, GSI-I combined with BTZ significantly inhibited tumor growth. **B.** Quantitative analysis of tumor volume indicates that the combination treatment was superior to treatment with a single drug in suppressing tumor growth. For instance, compared with control mice, combined treatment with GSI-I and BTZ significantly limited tumor growth starting from day 9 after treatment (*P* < 0.05). In contrast, inhibitory effects were observed at day 11 for BTZ (*P* < 0.05) and day 13 for GSI-I (*P* < 0.01). With the progress of the experiment at days 11 through day 15, the regression of tumor volumes was more evident after combined treatment with GSI-I and BTZ. At day 13, combined treatment was associated with more significant inhibitory effects than treatment with GSI-I alone, which became more pronounced at day 15. In addition, at day 15, the combined regimen was more effective than treatment with BTZ alone (*: *P* < 0.05 vs. control; †: *P* < 0.001 vs. control; ‡: *P* < 0.0001 vs. control and *P* < 0.05 vs. GSI-I; ¥: *P* < 0.0001 vs. control, *P* < 0.01 vs. GSI-I, *P* < 0.05 vs. BTZ). **C.** Quantitative analysis of TUNEL staining performed on tumor tissues collected at necropsy shows that there was a significant increase in apoptotic cells in tumors tissues from mice treated with GSI-I or BTZ alone compared with tumors collected from control mice. Nonetheless, the highest percentage of apoptotic cells were detected in the mice simultaneously treated with GSI-I and BTZ, and this percentage was not only significantly higher than control tumors but also than tumors from mice treated with either GSI-I or BTZ (*: *P* < 0.0001 vs. control; †: *P* < 0.0001 vs. control, GSI-I, and BTZ). Data represent means ± SE. **D.** Photomicrographs show representative examples of tumor sections collected from a control, untreated mouse stained with H&E (left panel). In addition, representative examples of tumor tissues from the different treatment groups are shown after being stained with TUNEL technique. Tumor tissues from the mouse treated with combined GSI-I and BTZ show more apoptotic cells compared with mice treated with GSI-I or BTZ alone. Original magnification: ×400. E. IHC stain shows pronounced expression of Notch1 and pAKT in the lymphoma xenograft tumors from control mice. Single treatments by GSI-I or BTZ slightly decreased the expression of Notch1 and pAKT. Importantly, combined treatment with the two inhibitors abrogated the expression of Notch1 and pAKT proteins. The expression of p-p38 MAPK was not detected in control lymphoma tumors, whereas treatment with GSI-I or BTZ slightly enhanced its expression. Notably, combined treatment with GSI-I and BTZ significantly increased the expression of p-p38 MAPK in the lymphoma cells. Original magnification: ×400.

The therapeutic effects of GSI-I and BTZ on T-LPN cells were also evaluated *in vivo* in a nude mouse model. Subcutaneous injection of Jurkat cells into the flanks of nude mice resulted in tumors at the site of injection. While therapy with either GSI-I or BTZ was able to delay tumor growth to some extent, the combination regimen induced remarkable regression of the tumor size. Representative images from the different mice groups are depicted in Fig. 6A, with dissected lymphoma tumors shown to illustrate the tumor sizes in the different groups. Quantitative measurement of tumor volume indicated that, as early as day 9, the effects of the combination treatment were more significant compared to the control group (Fig. 6B). Importantly, mice treated with combined GSI-I and BTZ exhibited tumor sizes that were markedly smaller than GSI-I or BTZ alone (Fig. 6B).

Assessment of apoptosis by the TUNEL staining was performed in tumor tissues collected at necropsy. Compared with the control and single-agent treatments, combining GSI-I and BTZ was associated with increased numbers of apoptotic cells (Fig. 6C). Representative examples of H&E-stained tumors as well as tissues stained with TUNEL technique are illustrated in Fig. 6D.

We also used immunohistochemical staining (IHC) to probe the xenograft T-LPN tumors with antibodies against Notch1, pAKT, and p-p38 MAPK proteins. In the control group, the expression of Notch1 was detected with a variable degree of intensity in most of the tumor cells. The intensity and frequency of the expression of Notch1 decreased significantly in the mice treated with GSI-I or BTZ alone. However, combining GSI-I and BTZ was associated with total lack of expression of Notch1 in the corresponding tumor tissues (Fig. 6E). pAKT showed a pattern of expression similar to Notch1, as it became largely absent in T-LPN tissues from mice simultaneously treated with GSI-I and BTZ. At the other hand, the expression of p-p38 MAPK was not present in tumors from control mice. Whereas the expression of p-p38 MAPK slightly increased after treatment with GSI-I or BTZ alone, this expression dramatically increased in mice simultaneously treated with the two inhibitors (Fig. 6E).

## DISCUSSION

Notch1 signaling has multiple pleiotropic effects during embryonic and postnatal development including cell fate determination and homeostasis [29–31]. Aberrant activation of Notch1 signaling has been linked to T-LPN [16, 32–36]. Although Notch1 signaling has recently become the focus of significant research, its precise role in T-LPN pathogenesis is not completely characterized. Our study provides novel evidence to support that the γ-secretase inhibitor GSI-I, which blocks Notch1 signaling, and the proteasome inhibitor BTZ cooperate together to effectively inhibit T-LPN *in vitro* and *in vivo*. Although treatment with GSI-I or BTZ alone induced anti-tumorigenic effects in the cell lines and primary human T-LPN cells, the effects of combining low concentrations of each inhibitor were significantly more pronounced. *NOTCH1* mutations were identified in 3 of the 4 tested patients, all with TLL. Individual response of the 4 patients to GSI-I and BTZ was similar, which suggests that the response to these inhibitors was not related to the *NOTCH1* mutation status. However, it is important to state that the patient population included in our study is very small and the effects of the inhibitors were analyzed *in vitro* using isolated TLL cells. Thus, definitive conclusions can not be firmly established.

Isobologram studies showed that GSI-I in conjunction with BTZ inhibited the proliferation of T-LPN cells in a synergistic fashion. We initially noticed remarkable variability in the sensitivities of the different cell lines to the effects of GSI-I or BTZ. For instance, MOLT-4 cells demonstrated higher susceptibility to the effects of GSI-I and BTZ on cell proliferation than the other cell lines. Therefore, in order to collect meaningful data to generate the isobolographic curves, we opted to use a different drug concentration range for each cell line. Thereafter, a concentration equivalent to IC_30_ specific for each cell line was used in the *in vitro* experiments.

Indeed, different cell lines used in this study demonstrated notable variability in their response to GSI-I and BTZ. Using morphological criteria, the Jurkat and H9 cells showed the lowest and highest number of apoptotic cells, respectively. Furthermore, MOLT-4 showed highest number of apoptotic cells using flow cytometry and Annexin V staining. Although the exact mechanisms underlying this variability are not clear, some important factors need to be considered. The differences among apoptosis detection methods could lead, at least partially, to the variability in the results among the different cell lines. In addition, despite the fact that these cell lines are classical representative of T-LPN, they carry heterogeneous biological traits, which stems to the fact that TLL and MF/SS have remarkably distinct clinicopathological characteristics. For instance, Jurkat and MOLT-4 cell lines are composed of immature lymphoid cells (blasts) that were developed from TLL. In contrast, the H9 and HuT 78 cell lines represent MF/SS T-cell lymphoma cells that are immunophenotypically mature. Considering the known inherent cell lines variability, our findings could resemble the clinical setting where different patients demonstrate variable responses to targeted therapy. Our results are also consistent with previous studies that showed that BTZ and γ-secretase inhibitors induce variable responses in different cell lines from the same type of cancer [37–39].

Our data support synergistic collaboration between GSI-I and BTZ in T-LPN. Although the exact explanations for this interesting phenomenon are not completely known, a recent study in T-LPN cells including Jurkat and MOLT-4 showed that BTZ induced negative regulatory effects on Notch1 signaling via repression of the transcription of *NOTCH1* gene and, as a result, downregulation of its effectors [40]. Considering that GSI-I induces its inhibitory effects on Notch1 via inhibition of the release of NICD, which also leads to suppression of *NOTCH1*-mediated transcriptional activation of target genes, combining GSI-I with BTZ will most likely enhance their inhibitory effects on Notch1 signaling. In a similar fashion, GSI-I has also been shown to block proteasomal activity with potency comparable to BTZ, which could further explain the synergistic collaboration between the two inhibitors [41].

A main hurdle for using GSI-I or BTZ as single agents was dose-limiting toxicities that caused deleterious side effects [20, 21, 28, 42]. The studies investigating combining γ-secretase and proteasome inhibitors are few, with one recent report demonstrating that combining low concentrations of GSI-XII and BTZ potentiates the *in vitro* effects of the single agent [39]. It is important to highlight that our study is the first to extensively investigate the *in vitro* and *in vivo* effects of combining low concentrations of GSI-I and BTZ in T-LPN.

Although treatment with GSI-I or BTZ alone induced apoptosis in T-LPN cells and decreased their proliferation, these effects were more pronounced when GSI-I and BTZ were simultaneously utilized to treat the cells. Consistent with the occurrence of apoptotic cell death, GSI-I or BTZ alone induced PARP and caspase 3 cleavages, and decreased BCL-2 and BCL-xL levels, and these effects increased significantly with the combined treatment. In addition to the *in vitro* evidence, combined treatment with GSI-I and BTZ also sensitized T-LPN tumor cells to *in vivo* apoptosis and halted tumor growth in nude mice more efficaciously than treatment with a single agent.

After treating T-LPN cells by GSI-I or BTZ, Notch1 and NICD proteins were slightly decreased. Importantly, combined treatment was associated with more remarkable decrease in Notch1 and NICD levels. Notch1 facilitates direct and indirect targeting of genes particularly oncogenes such as *MYC* [5, 43, 44]. The NOTCH1-MYC loop acts as a critical driver of cell growth and anabolism in T-LPN [45]. Another downstream target of Notch1 is Hes1, which contributes to tumor progression [46]. Previous studies showed that inhibition of Notch1 via suppression of γ-secretase activity reduces c-Myc and Hes1 levels, which was associated with decreased tumor cell proliferation [47, 48]. Similarly, in our study, treatment of the T-LPN cells with GSI-I decreased c-Myc and Hes1 levels, and decreased cellular proliferation. Importantly, the effects of GSI-I on c-Myc and Hes1 were much more potentiated when it was combined with BTZ.

The AKT and ERK pathway play important roles in maintaining the survival of T-LPN, at least partially, through interactions with Notch1 [49–52]. Our study shows that GSI-I alone did not induce any notable effects on AKT phosphorylated at Ser^473^ in Jurkat cells, and induced minimal decrease in the phosphorylation levels of this serine residue in HuT 78 cells. These results are consistent with the recent report that showed that inhibition of Notch1 by GSI-IX increased the phosphorylation of AKT at Thr^308^ with no effects on Ser^473^ [53]. In our study, in addition to GSI-I, treating Jurkat and HuT 78 cells with BTZ induced a slight decrease in pAKT-Ser^473^. Combined treatment with GSI-I and BTZ potentiated their effects and caused marked decrease in pAKT-Ser^473^. Although GSI-I or BTZ alone induced moderate or mild decrease, respectively, in pERK-Thr^202^/Tyr^204^, the combined effects of the two inhibitors were much more pronounced as the levels of pERK decreased dramatically after treatment. Thus, combined abrogation of pAKT and pERK appears to be an important mechanism underlying drug synergy between GSI-I and BTZ in T-LPN.

JNK and p38 MAPK are also involved in the regulation of apoptosis, cell cycle, differentiation, and stress response of cancer cells including T-LPN [54–56]. Recent studies suggested that JNK and p38 MAPK have dual roles by promoting cancer cell death or survival depending on the cell type, nature of the death stimuli, and the contribution of other interacting survival molecules [56, 57]. Furthermore, it was previously shown in lymphoid and myeloid cells that apoptosis and cell death induced by BTZ or AKT inhibitors were associated with increased p38 MAPK and JNK phosphorylation [56, 58]. These findings are consistent with our results demonstrating that T-LPN cell death induced by combined treatment with GSI-I and BTZ was associated with remarkable increase in pJNK. Furthermore, treatment with a single agent increased p-p38 MAPK levels, and combining GSI-I and BTZ induced a very slight additional increase in p-p38 MAPK levels.

NF-κB is constitutively activated in T-LPN, and the functional interactions between Notch1 and NF-κB have been extensively studied [59, 60]. Notch1 activation leads to the phosphorylation of IκBα and subsequent activation of NF-κB, mediated by IκBα kinase signalosome [61]. In T-LPN, generally IκBα is readily degraded upon activation of Notch1 signaling [62]. The proteasome inhibitor BTZ was expected to antagonize the degradation of IκBα and increase its levels. Although, there was no direct increase in IκBα basal levels in our study, BTZ indirectly increased basal IκBα levels through downregulation of pIκBα, which led to increased IκBα-to-pIκBα ratio. Notably, this increase was modest, yet it became much more pronounced when BTZ was combined with GSI-I, attesting the synergistic effects of the two inhibitors. Although we cannot entirely explain the lack of a more robust effect of BTZ alone on IκBα levels, important factors might have led to this unexpected finding including: 1) type of cells; Jurkat (leukemic blasts) vs. HuT 78 (malignant mature lymphoma cells); 2) concentration of BTZ; and 3) duration of treatment by BTZ. Similar to our results, a previously published study showed lack of significant changes in total IκBα at 24 h after treating Jurkat cells with 10 nM BTZ; a concentration similar to the one used in our study. Only a slight increment in IκBα was noted at 48 h [63]. In contrast to the anticipated BTZ-induced upregulation of IκBα, evidence in plasma cell myeloma cell lines and primary cells from patients suggests that BTZ could also significantly downregulate IκBα expression, which subsequently triggers NF-κB activation [64]. Despite these effects on IκBα/NF-κB, BTZ was able to effectively cause cytotoxicity in the myeloma cells. Collectively, these results suggest that BTZ-induced antitumor activity may be mediated dependently or independently from the NF-κB system. At least from our data, combined targeting of Notch1 and proteasome appears to upregulate IκBα expression more than targeting the proteasome system alone.

Using GSI-I or BTZ as single agents did not affect the nuclear levels of expression of p65 subunit of NF-κB in T-LPN cells. In contrast, the nuclear expression of p65 decreased significantly after the combined treatment, which indirectly implies that NF-κB became predominantly sequestered within the cytoplasmic compartment. Furthermore, pIKKα/β levels were dramatically decreased after the combined treatment with GSI-I and BTZ than when one inhibitor was used alone. This indicates that combining GSI-I and BTZ triggers repression of NF-κB activity, which contributed to apoptosis and cell death in T-LPN.

To analyze the individual contributions of survival pathways to the synergistic effects of GSI-I and BTZ, we elected to focus on AKT and p38 MAPK, which supports and suppresses the survival of T-LPN, respectively. Treatment with the selective AKT inhibitor MK-2206 in conjunction with GSI-I and BTZ decreased the viability of Jurkat and HuT 78 cells more than the combined treatment with GSI-I and BTZ alone. Notably, MK-2206 failed to induce effects on the viability of T-LPN cells at low concentrations and marginally decreased their viability at higher concentrations. These findings suggest that combined targeting of Notch1 and proteasome perhaps has superior negative impact on T-LPN than targeting AKT. At the other hand, selective inhibition of p38 MAPK by SB203580 alone, at two different concentrations, did not affect the viability of T-LPN cells. In contrast, GSI-I and BTZ decreased T-LPN cell viability significantly, yet SB203580 was able to partially rescue these cells when it was simultaneously used with GSI-I and BTZ. These findings suggest that p38 MAPK is most likely enhancing the inhibitory effects of GSI-I and BTZ on T-LPN.

We also studied the effects of GSI-I and BTZ, alone or in combination, *in vivo* using nude mice with Jurkat cell-driven xenografts. Combining low doses of GSI-I (5 mg/kg) and BTZ (60 μg/kg) suppressed the growth of Jurkat cell xenografts more efficiently than either drug alone. In addition, apoptosis was more evident in Jurkat tumors collected from mice treated simultaneously with GSI-I and BTZ than mice treated with either drug alone. It is important to note that the apoptosis rate observed in the Jurkat cell lymphoma tumor xenografts was lower than the one detected *in vitro* in Jurkat cells. It is possible that the *in vivo* inhibitory effects of GSI-I and BTZ were related to suppression of lymphoma cells growth via hindering cell proliferation than inducing apoptosis. Although the exact explanations underlying the discrepancies between *in vitro* and *in vivo* findings are not totally known, important factors need to be taken in consideration including the biological differences between cell line suspensions that contain a pure population of Jurkat cells and the lymphoma tumor xenografts that contain stromal cells, which may protect the lymphoma cells from apoptosis. Moreover, differences in sensitivities exist between flow cytometry, which was used to measure apoptosis in cell line suspensions, and the TUNEL assay that was utilized to identify apoptotic cells in formalin-fixed and paraffin-embedded tissue sections.

To further analyze the effects of GSI-I and BTZ, Jurkat cell lymphoma xenografts were probed using IHC and specific antibodies against Notch1, pAKT, and p-p38 MAPK proteins. GSI-I or BTZ alone decreased the expression of Notch1 protein, but the effects of combined treatment on Notch1 expression was much more pronounced. Similarly, the single treatments decreased pAKT expression, and combining GSI-I and BTZ abrogated this expression. Whereas lymphoma tissues from control mice demonstrated lack of expression of p-p38 MAPK, GSI-I or BTZ alone slightly increased p-p38 MAPK. Importantly, combining GSI-I and BTZ enhanced significantly the expression of p-p38 MAPK. Despite that the IHC findings in general were consistent with the *in vitro* Western blot data, variability was noted. For example, the increase in the expression of p-p38 MAPK after treating the cells with GSI-I or BTZ alone was more pronounced *in vitro* than the *in vivo.* Similar to the possible explanations for the variability in apoptosis levels addressed above, the variability in the changes in survival proteins between *in vitro* and *in vivo* experimental approaches could be due to the differences in the nature of the specimens as well as the protein detection methods.

It is important to highlight that apparent toxicity was not observed in the nude mice because of the relatively low doses of GSI-I and BTZ that were intermittently administered in our model. Indeed, the low doses of GSI-I and BTZ used in our study were less than the doses currently utilized in clinical trials (https://clinicaltrials.gov/ct2/show/NCT00878189) that enroll T-LPN patients [65, 66].

Taken together, combined targeting of Notch1 and proteasome by GSI-I and BTZ, respectively, causes synergistic tumor suppression of T-LPN. The combined effects of GSI-I and BTZ are significantly more pronounced than the isolated effects of GSI-I or BTZ. Importantly, the findings observed *in vitro* were consistent with those observed *in vivo*, which supports the efficacy of this approach and provides legitimate rationale for clinical utilization to treat T-LPN patients.

## MATERIALS AND METHODS

### Cell lines and reagents

TLL (Jurkat and MOLT-4) and MF/SS (HuT 78, and H9) cell lines were purchased from American Type Culture Collection (Bethesda, MD). Normal human peripheral blood CD3^+^ pan-T lymphocytes were purchased from StemCell Technologies (Vancouver, BC, Canada; Catalog number: PB009–1F). Cells were maintained in RPMI-1640 medium (HyClone; Thermo Fisher Scientific, Waltham, MA), supplemented with 10% heat-inactivated fetal bovine serum, 2 mM glutamine, 100 U/mL penicillin, 100 μg/mL streptomycin in a humidified atmosphere of 95% air and 5% CO_2_ at 37°C. The γ-secretase inhibitor GSI-I (Z-Leu-Leu-Nle-CHO) was purchased from Merck Calbiochem (San Diego, CA). The proteasome inhibitor BTZ, the AKT inhibitor MK-2206, and the p-p38 MAPK inhibitor SB203580 were obtained from Selleckchem (Houston, TX). A concentration-response curve was generated for each cell line as shown in Fig. 3, then, a concentration equivalent to IC_30_ specific for each cell line was used in the *in vitro* experiments.

### Cell viability by MTS assay

Changes in cell viability were measured as previously described [67, 68]. Cells were treated with GSI-I and BTZ alone or in combination, and with or without AKT/p38 MAPK inhibitors at different concentrations in 96-well plates. In the case of inhibitors, cells were pre-treated over night with specific inhibitors before adding drugs. After 48 h, 20 μL of the MTS reagent (Promega, Madison, WI) was added into each well. Samples were incubated at 37°C for 1–4 h and the absorbance was measured at 490 nm by spectrophotometry.

### Cell proliferation by BrdU assay

Cell proliferation was measured by using the BrdU cell proliferation kit (X1327K1, Exalpha Biologicals, Shirley, MD) as previously described [67, 68]. After treatment for 24 h, cells were plated at a concentration of 2 × 10^5^ cells/mL in 96 well plates. Twenty-μL of BrdU (diluted 1:500) was added and incubated overnight. The Fixing Solution was then added and plates subjected to washing. Thereafter, 100 μL/well of anti-BrdU monoclonal antibody were added, followed by 100 μL/well peroxidase goat anti-mouse IgG conjugate (diluted 1:2000). The plates were again washed, and 100 μL/well of TMB substrate were added. Stop solution (50 μL) was added and plates were read in an ELISA plate reader (450/595 nm).

### Anchorage-independent colony formation assay

Cells were plated in a methylcellulose-based medium (Methocult H4230, Stemcell Technologies) and mixed in RPMI-1640 medium at a ratio of 1:4 (v/v) [67, 68]. Harvested cells were mixed in a 1:10 (v/v) ratio with methylcellulose in 15 mL conical tubes that were then inverted gently. Thereafter, the contents were poured into 24-well plates, and incubated at 37°C in a 5% CO_2_ incubator for 5 days. Then, p-iodonitrotetrazolium violet was added and incubated overnight. Colonies were visualized using the FluorChem 8800 imaging system (Alpha Innotech, San Leandro, CA).

### Apoptosis detection

*In vitro* apoptosis was evaluated using a standard Kit (556547, BD Biosciences, San Jose, CA) as previously described [67, 68]. Cells were dual-stained with Annexin V and PI and the fluorescent intensity was measured by flow cytometry (BD FACSCalibur system). Percentage of apoptotic cells was quantified using FlowJo software (BD Biosciences). In addition, apoptosis was detected using morphometric analysis. Briefly, cytospins were prepared by centrifugation and stained with Wright-Giemsa stain. Thereafter, morphologic features consistent with apoptosis were evaluated using light microscopy.

### Isobolographic analysis

Based on concentration–response curves obtained from the *in vitro* treatments of cell lines, three isoeffect curves were generated by using isobolograms to determine the synergistic vs. additive vs. antagonistic effects of GSI-I and BTZ [69]. Concentration-dependent effects were calculated using MS-Excel (Microsoft; Redmond, WA) for one drug while keeping constant concentrations for the other.

### Patient samples

Experiments in human samples were performed in accordance with the Declaration of Helsinki and after approval of the Institutional Review Board. Primary samples from 8 T-LPN patients were included in this study. Cells were isolated and treated with GSI-I or BTZ alone, or in combination. Post-treatment effects were analyzed using MTS for cell viability and Giemsa stain for morphological changes associated with apoptosis.

### Antibodies

Antibodies purchased from Santa Cruz Biotechnology (Dallas, TX) included caspase-3 (sc-7272), BCL-2 (sc-7382), ERK (sc-94), and GAPDH (sc-25778); from Cell Signaling Technology (Danvers, MA) were cleaved caspase-3 (9661S), cleaved PARP (5625S), PARP (9542P), pAKT (Ser^473^, 4051), AKT (9272), pERK (Thr^202^/Tyr^204^, 4370S), pIKKα/β (Ser^176/177^, 2078S), IKKα (2682S), pIκBα (Ser^32/36^, 9246S), IκBα (4814S), p65 (6956S), Notch1 (3608S), cleaved Notch1 (NICD, 4147S), c-Myc (5605S), Hes1 (11988S), pJNK (4668S), JNK (9252), p-p38 (Thr^180^/Tyr^182^, 4511S), p38 (8690S), and lamin B2 (9622); and from Zymed Laboratories (South San Francisco, CA) was BCL-xL (18–0217).

### Western blotting

Cells were lysed in lysis buffer (25 mM HEPES [pH 7.7], 1.5 mM MgCl_2_, 400 mM NaCl, 2 mM EDTA, 0.5% Triton X-100, 3 mM DTT, 0.1 mM PMSF), with phosphatase inhibitor (20 mM β-GP, 1 mM Na_3_VO_4_), and protease inhibitor cocktails (10 μg/ml leupeptin, 2 μg/ml pepstatin, 50 μg/ml antipain, 1× benzamidine, 2 μg/ml aprotinin, 20 μg/ml chymostatin) (Roche; Indianapolis, IN) as previously described [67, 68]. Fractionation was performed using nuclear/cytosol fractionation kit (BioVision, Milpitas, CA). Protein extracts (50 μg) were loaded onto 8–12% polyacrylamide gel containing SDS, electrophoresed, and transferred to a polyvinylidene fluoride membrane. The membranes were blocked with 5% non-fat dried milk in Tris-buffered saline/0.1% Tween-20 and incubated overnight at 4°C with the desired primary antibody, followed by a matched horseradish peroxidase-linked secondary antibody. The immunocomplexes were visualized using a chemiluminescence horseradish peroxidase kit. GAPDH and lamin B2 antibodies were used as the loading controls for whole cell and nuclear proteins, respectively.

### Murine model

Experiments in mice were approved by the Animal Ethical Committee at Nantong University. Nude mice (5–6 weeks old; 8 mice in each group; Shanghai Laboratory Animal Center, Shanghai, China) were injected subcutaneously into the right flank with 4 × 10^7^ Jurkat cells. Then, treatments were started at day 21. Control mice received PBS, while the other 3 groups received GSI-I (i.p.: 5 mg/kg/day over a period of 14 days; two cycles; 5 days on and 2 days off), BTZ (i.p.: 60 μg/kg once per day for 14 days), or the combination treatment. Tumor volumes were measured every 2 days and calculated using the formula 0.5 × a × b^2^ (a: length; b: width). Animals were sacrificed after 14 days by CO_2_ asphyxiation and cervical dislocation according to the animal protocol. Tumors were fixed in 10% buffered formalin and then embedded in paraffin.

### Terminal deoxytransferase-dUTP nick-end labeling (TUNEL) assay

*In situ* cellular apoptosis was evaluated by detection of fragmented DNA on deparaffinized formalin-fixed sections from mice lymphoma xenografts (5.0 μm). At least 500 cells in each of three different fields were evaluated using light microscopy.

### IHC of tumor xenografts from nude mice

IHC was performed on formalin-fixed and paraffin-embedded xenograft tumor tissue sections prepared using standard techniques [67]. Antibodies used were purchased from Cell Signaling and included: p-p38 MAPK (4511S; 1:25), Notch1 (3608S; 1:50) and pAKT (Ser^473^, 4060; 1:50). Photomicrographs were taken using a Nikon Microphot FXA microscope (Nikon Instruments, Melville, NY) and an Olympus DP70 camera (Olympus America, Melville, NY).

### Statistical analysis

Statistical analyses for the *in vitro* and *in vivo* studies were performed by one-way ANOVA, and *P* < 0.05 was considered to be statistically significant (GraphPad PRISM software; GraphPad Software Inc., San Diego, CA).

## References

[1] Bazarbachi A, Ghez D, Lepelletier Y, Nasr R, de The H, El-Sabban ME, Hermine O (2004). New therapeutic approaches for adult T-cell leukaemia. Lancet Oncol.

[2] Taylor GP, Matsuoka M (2005). Natural history of adult T-cell leukemia/lymphoma and approaches to therapy. Oncogene.

[3] Ferrando AA (2009). The role of NOTCH1 signaling in T-ALL. Hematology Am Soc Hematol Educ Program.

[4] Haruki N, Kawaguchi KS, Eichenberger S, Massion PP, Olson S, Gonzalez A, Carbone DP, Dang TP (2005). Dominant-negative Notch3 receptor inhibits mitogen-activated protein kinase pathway and the growth of human lung cancers. Cancer Res.

[5] Palomero T, Lim WK, Odom DT, Sulis ML, Real PJ, Margolin A, Barnes KC, O'Neil J, Neuberg D, Weng AP, Aster JC, Sigaux F, Soulier J (2006). NOTCH1 directly regulates c-MYC and activates a feed-forward-loop transcriptional network promoting leukemic cell growth. Proc Natl Acad Sci U S A.

[6] Pece S, Serresi M, Santolini E, Capra M, Hulleman E, Galimberti V, Zurrida S, Maisonneuve P, Viale G, Di Fiore PP (2004). Loss of negative regulation by Numb over Notch is relevant to human breast carcinogenesis. J Cell Biol.

[7] Purow BW, Haque RM, Noel MW, Su Q, Burdick MJ, Lee J, Sundaresan T, Pastorino S, Park JK, Mikolaenko I, Maric D, Eberhart CG, Fine HA (2005). Expression of Notch-1 and its ligands, Delta-like-1 and Jagged-1, is critical for glioma cell survival and proliferation. Cancer Res.

[8] Grabher C, von Boehmer H, Look AT (2006). Notch 1 activation in the molecular pathogenesis of T-cell acute lymphoblastic leukaemia. Nat Rev Cancer.

[9] Guruharsha KG, Kankel MW, Artavanis-Tsakonas S (2012). The Notch signalling system: recent insights into the complexity of a conserved pathway. Nat Rev Genet.

[10] Kopan R, Ilagan MX (2009). The canonical Notch signaling pathway: unfolding the activation mechanism. Cell.

[11] Bush G, diSibio G, Miyamoto A, Denault JB, Leduc R, Weinmaster G (2001). Ligand-induced signaling in the absence of furin processing of Notch1. Dev Biol.

[12] Munoz-Descalzo S, Tkocz K, Balayo T, Arias AM (2011). Modulation of the ligand-independent traffic of Notch by Axin and Apc contributes to the activation of Armadillo in Drosophila. Development.

[13] Ordentlich P, Lin A, Shen CP, Blaumueller C, Matsuno K, Artavanis-Tsakonas S, Kadesch T (1998). Notch inhibition of E47 supports the existence of a novel signaling pathway. Mol Cell Biol.

[14] Shin HM, Minter LM, Cho OH, Gottipati S, Fauq AH, Golde TE, Sonenshein GE, Osborne BA (2006). Notch1 augments NF-kappaB activity by facilitating its nuclear retention. EMBO J.

[15] Ellisen LW, Bird J, West DC, Soreng AL, Reynolds TC, Smith SD, Sklar J (1991). TAN-1, the human homolog of the Drosophila notch gene, is broken by chromosomal translocations in T lymphoblastic neoplasms. Cell.

[16] Weng AP, Ferrando AA, Lee W, Morris JPt, Silverman LB, Sanchez-Irizarry C, Blacklow SC, Look AT, Aster JC (2004). Activating mutations of NOTCH1 in human T cell acute lymphoblastic leukemia. Science.

[17] Kamstrup MR, Gjerdrum LM, Biskup E, Lauenborg BT, Ralfkiaer E, Woetmann A, Odum N, Gniadecki R (2010). Notch1 as a potential therapeutic target in cutaneous T-cell lymphoma. Blood.

[18] Kamstrup MR, Ralfkiaer E, Skovgaard GL, Gniadecki R (2008). Potential involvement of Notch1 signalling in the pathogenesis of primary cutaneous CD30-positive lymphoproliferative disorders. Br J Dermatol.

[19] Palomero T, Barnes KC, Real PJ, Glade Bender JL, Sulis ML, Murty VV, Colovai AI, Balbin M, Ferrando AA (2006). CUTLL1, a novel human T-cell lymphoma cell line with t(7, 9) rearrangement, aberrant NOTCH1 activation and high sensitivity to gamma-secretase inhibitors. Leukemia.

[20] Milano J, McKay J, Dagenais C, Foster-Brown L, Pognan F, Gadient R, Jacobs RT, Zacco A, Greenberg B, Ciaccio PJ (2004). Modulation of notch processing by gamma-secretase inhibitors causes intestinal goblet cell metaplasia and induction of genes known to specify gut secretory lineage differentiation. Toxicol Sci.

[21] Staal FJ, Langerak AW (2008). Signaling pathways involved in the development of T-cell acute lymphoblastic leukemia. Haematologica.

[22] Goy A, Younes A, McLaughlin P, Pro B, Romaguera JE, Hagemeister F, Fayad L, Dang NH, Samaniego F, Wang M, Broglio K, Samuels B, Gilles F (2005). Phase II study of proteasome inhibitor bortezomib in relapsed or refractory B-cell non-Hodgkin's lymphoma. J Clin Oncol.

[23] Masdehors P, Omura S, Merle-Beral H, Mentz F, Cosset JM, Dumont J, Magdelenat H, Delic J (1999). Increased sensitivity of CLL-derived lymphocytes to apoptotic death activation by the proteasome-specific inhibitor lactacystin. Br J Haematol.

[24] O'Connor OA, Wright J, Moskowitz C, Muzzy J, MacGregor-Cortelli B, Stubblefield M, Straus D, Portlock C, Hamlin P, Choi E, Dumetrescu O, Esseltine D, Trehu E (2005). Phase II clinical experience with the novel proteasome inhibitor bortezomib in patients with indolent non-Hodgkin's lymphoma and mantle cell lymphoma. J Clin Oncol.

[25] Orlowski RZ, Eswara JR, Lafond-Walker A, Grever MR, Orlowski M, Dang CV (1998). Tumor growth inhibition induced in a murine model of human Burkitt's lymphoma by a proteasome inhibitor. Cancer Res.

[26] Tan C, Waldmann TA (2002). Proteasome inhibitor PS-341, a potential therapeutic agent for adult T-cell leukemia. Cancer Res.

[27] Kane RC, Farrell AT, Sridhara R, Pazdur R (2006). United States Food and Drug Administration approval summary: bortezomib for the treatment of progressive multiple myeloma after one prior therapy. Clin Cancer Res.

[28] Field-Smith A, Morgan GJ, Davies FE (2006). Bortezomib (Velcadetrade mark) in the Treatment of Multiple Myeloma. Ther Clin Risk Manag.

[29] Aster JC, Pear WS, Blacklow SC (2008). Notch signaling in leukemia. Annu Rev Pathol.

[30] Lin YW, Nichols RA, Letterio JJ, Aplan PD (2006). Notch1 mutations are important for leukemic transformation in murine models of precursor-T leukemia/lymphoma. Blood.

[31] Shih Ie M, Wang TL (2007). Notch signaling, gamma-secretase inhibitors, and cancer therapy. Cancer Res.

[32] Breit S, Stanulla M, Flohr T, Schrappe M, Ludwig WD, Tolle G, Happich M, Muckenthaler MU, Kulozik AE (2006). Activating NOTCH1 mutations predict favorable early treatment response and long-term outcome in childhood precursor T-cell lymphoblastic leukemia. Blood.

[33] Jundt F, Anagnostopoulos I, Forster R, Mathas S, Stein H, Dorken B (2002). Activated Notch1 signaling promotes tumor cell proliferation and survival in Hodgkin and anaplastic large cell lymphoma. Blood.

[34] Pinnix CC, Lee JT, Liu ZJ, McDaid R, Balint K, Beverly LJ, Brafford PA, Xiao M, Himes B, Zabierowski SE, Yashiro-Ohtani Y, Nathanson KL, Bengston A (2009). Active Notch1 confers a transformed phenotype to primary human melanocytes. Cancer Res.

[35] Santagata S, Demichelis F, Riva A, Varambally S, Hofer MD, Kutok JL, Kim R, Tang J, Montie JE, Chinnaiyan AM, Rubin MA, Aster JC (2004). JAGGED1 expression is associated with prostate cancer metastasis and recurrence. Cancer Res.

[36] Zagouras P, Stifani S, Blaumueller CM, Carcangiu ML, Artavanis-Tsakonas S (1995). Alterations in Notch signaling in neoplastic lesions of the human cervix. Proc Natl Acad Sci U S A.

[37] Aleksic T, Feller SM (2008). Gamma-secretase inhibition combined with platinum compounds enhances cell death in a large subset of colorectal cancer cells. Cell Commun Signal.

[38] Codony-Servat J, Tapia MA, Bosch M, Oliva C, Domingo-Domenech J, Mellado B, Rolfe M, Ross JS, Gascon P, Rovira A, Albanell J (2006). Differential cellular and molecular effects of bortezomib, a proteasome inhibitor, in human breast cancer cells. Mol Cancer Ther.

[39] Williams S, Pettaway C, Song R, Papandreou C, Logothetis C, McConkey DJ (2003). Differential effects of the proteasome inhibitor bortezomib on apoptosis and angiogenesis in human prostate tumor xenografts. Mol Cancer Ther.

[40] Koyama D, Kikuchi J, Hiraoka N, Wada T, Kurosawa H, Chiba S, Furukawa Y (2014). Proteasome inhibitors exert cytotoxicity and increase chemosensitivity via transcriptional repression of Notch1 in T-cell acute lymphoblastic leukemia. Leukemia.

[41] Biskup E, Kamstrup MR, Manfe V, Gniadecki R (2013). Proteasome inhibition as a novel mechanism of the proapoptotic activity of gamma-secretase inhibitor I in cutaneous T-cell lymphoma. Br J Dermatol.

[42] Chen F, Pisklakova A, Li M, Baz R, Sullivan DM, Nefedova Y (2011). Gamma-secretase inhibitor enhances the cytotoxic effect of bortezomib in multiple myeloma. Cell Oncol (Dordr).

[43] Sharma VM, Calvo JA, Draheim KM, Cunningham LA, Hermance N, Beverly L, Krishnamoorthy V, Bhasin M, Capobianco AJ, Kelliher MA (2006). Notch1 contributes to mouse T-cell leukemia by directly inducing the expression of c-myc. Mol Cell Biol.

[44] Weng AP, Millholland JM, Yashiro-Ohtani Y, Arcangeli ML, Lau A, Wai C, Del Bianco C, Rodriguez CG, Sai H, Tobias J, Li Y, Wolfe MS, Shachaf C (2006). c-Myc is an important direct target of Notch1 in T-cell acute lymphoblastic leukemia/lymphoma. Genes Dev.

[45] Tzoneva G, Ferrando AA (2012). Recent advances on NOTCH signaling in T-ALL. Curr Top Microbiol Immunol.

[46] Moriyama M, Osawa M, Mak SS, Ohtsuka T, Yamamoto N, Han H, Delmas V, Kageyama R, Beermann F, Larue L, Nishikawa S (2006). Notch signaling via Hes1 transcription factor maintains survival of melanoblasts and melanocyte stem cells. J Cell Biol.

[47] Wang M, Wu L, Wang L, Xin X (2010). Down-regulation of Notch1 by gamma-secretase inhibition contributes to cell growth inhibition and apoptosis in ovarian cancer cells A2780. Biochem Biophys Res Commun.

[48] Weng AP, Millholland JM, Yashiro-Ohtani Y, Arcangeli ML, Lau A, Wai C, del Bianco C, Rodriguez CG, Sai H, Tobias J (2006). c-Myc is an important direct target of Notch1 in T-cell acute lymphoblastic leukemia/lymphoma. Genes & development.

[49] Dail M, Wong J, Lawrence J, O'Connor D, Nakitandwe J, Chen SC, Xu J, Lee LB, Akagi K, Li Q, Aster JC, Pear WS, Downing JR (2014). Loss of oncogenic Notch1 with resistance to a PI3K inhibitor in T-cell leukaemia. Nature.

[50] Hales EC, Taub JW, Matherly LH (2014). New insights into Notch1 regulation of the PI3K-AKT-mTOR1 signaling axis: targeted therapy of gamma-secretase inhibitor resistant T-cell acute lymphoblastic leukemia. Cell Signal.

[51] Barata JT, Silva A, Brandao JG, Nadler LM, Cardoso AA, Boussiotis VA (2004). Activation of PI3K is indispensable for interleukin 7-mediated viability, proliferation, glucose use, and growth of T cell acute lymphoblastic leukemia cells. J Exp Med.

[52] Palomero T, Ferrando A (2008). Oncogenic NOTCH1 control of MYC and PI3K: challenges and opportunities for anti-NOTCH1 therapy in T-cell acute lymphoblastic leukemias and lymphomas. Clin Cancer Res.

[53] Hales EC, Orr SM, Larson Gedman A, Taub JW, Matherly LH (2013). Notch1 receptor regulates AKT protein activation loop (Thr308) dephosphorylation through modulation of the PP2A phosphatase in phosphatase and tensin homolog (PTEN)-null T-cell acute lymphoblastic leukemia cells. J Biol Chem.

[54] Cuadrado A, Nebreda AR (2010). Mechanisms and functions of p38 MAPK signalling. Biochem J.

[55] Kyriakis JM, Banerjee P, Nikolakaki E, Dai T, Rubie EA, Ahmad MF, Avruch J, Woodgett JR (1994). The stress-activated protein kinase subfamily of c-Jun kinases. Nature.

[56] Chiarini F, Del Sole M, Mongiorgi S, Gaboardi GC, Cappellini A, Mantovani I, Follo MY, McCubrey JA, Martelli AM (2008). The novel Akt inhibitor, perifosine, induces caspase-dependent apoptosis and downregulates P-glycoprotein expression in multidrug-resistant human T-acute leukemia cells by a JNK-dependent mechanism. Leukemia.

[57] Sui X, Kong N, Ye L, Han W, Zhou J, Zhang Q, He C, Pan H (2014). p38 and JNK MAPK pathways control the balance of apoptosis and autophagy in response to chemotherapeutic agents. Cancer Lett.

[58] Yu C, Rahmani M, Dent P, Grant S (2004). The hierarchical relationship between MAPK signaling and ROS generation in human leukemia cells undergoing apoptosis in response to the proteasome inhibitor Bortezomib. Exp Cell Res.

[59] Mori N, Fujii M, Ikeda S, Yamada Y, Tomonaga M, Ballard DW, Yamamoto N (1999). Constitutive activation of NF-kappaB in primary adult T-cell leukemia cells. Blood.

[60] Rayet B, Gelinas C (1999). Aberrant rel/nfkb genes and activity in human cancer. Oncogene.

[61] Osipo C, Golde TE, Osborne BA, Miele LA (2008). Off the beaten pathway: the complex cross talk between Notch and NF-kappaB. Lab Invest.

[62] Martinez-Delgado B, Cuadros M, Honrado E, Ruiz de la Parte A, Roncador G, Alves J, Castrillo JM, Rivas C, Benitez J (2005). Differential expression of NF-kappaB pathway genes among peripheral T-cell lymphomas. Leukemia.

[63] Lu S, Chen Z, Yang J, Chen L, Gong S, Zhou H, Guo L, Wang J (2008). Overexpression of the PSMB5 gene contributes to bortezomib resistance in T-lymphoblastic lymphoma/leukemia cells derived from Jurkat line. Exp Hematol.

[64] Hideshima T, Ikeda H, Chauhan D, Okawa Y, Raje N, Podar K, Mitsiades C, Munshi NC, Richardson PG, Carrasco RD, Anderson KC (2009). Bortezomib induces canonical nuclear factor-kappaB activation in multiple myeloma cells. Blood.

[65] USFDA (2005). Guidance for Industry: Estimating the Maximum Safe Starting Dose in Adult Healthy Volunteer.

[66] Zinzani PL, Musuraca G, Tani M, Stefoni V, Marchi E, Fina M, Pellegrini C, Alinari L, Derenzini E, de Vivo A, Sabattini E, Pileri S, Baccarani M (2007). Phase II trial of proteasome inhibitor bortezomib in patients with relapsed or refractory cutaneous T-cell lymphoma. J Clin Oncol.

[67] George SK, Vishwamitra D, Manshouri R, Shi P, Amin HM (2014). The ALK inhibitor ASP3026 eradicates NPM-ALK(+) T-cell anaplastic large-cell lymphoma *in vitro* and in a systemic xenograft lymphoma model. Oncotarget.

[68] Shi P, Lai R, Lin Q, Iqbal AS, Young LC, Kwak LW, Ford RJ, Amin HM (2009). IGF-IR tyrosine kinase interacts with NPM-ALK oncogene to induce survival of T-cell ALK+ anaplastic large-cell lymphoma cells. Blood.

[69] Steel GG, Peckham MJ (1979). Exploitable mechanisms in combined radiotherapy-chemotherapy: the concept of additivity. Int J Radiat Oncol Biol Phys.

